# Cycle Checkpoint Abnormalities during Dementia: A Plausible Association with the Loss of Protection against Oxidative Stress in Alzheimer’s Disease

**DOI:** 10.1371/journal.pone.0068361

**Published:** 2013-07-05

**Authors:** Pavel Katsel, Weilun Tan, Peter Fam, Dushyant P. Purohit, Vahram Haroutunian

**Affiliations:** 1 Department of Psychiatry, The Mount Sinai School of Medicine, New York, New York, United States of America; 2 Department of Psychiatry, James J. Peters VA Medical Center, Bronx, New York, United States of America; 3 Department of Neuroscience, The Mount Sinai School of Medicine, New York, New York, United States of America; 4 Department of Pathology, The Mount Sinai School of Medicine, New York, New York, United States of America; Oregon Health & Science University, United States of America

## Abstract

**Background:**

Increasing evidence suggests an association between neuronal cell cycle (CCL) events and the processes that underlie neurodegeneration in Alzheimer’s disease (AD). Elevated levels of oxidative stress markers and mitochondrial dysfunction are also among early events in AD. Recent studies have reported the role of CCL checkpoint proteins and tumor suppressors, such as ATM and p53 in the control of glycolysis and oxidative metabolism in cancer, but their involvement in AD remains uncertain.

**Methods and Findings:**

In this postmortem study, we measured gene expression levels of eight CCL checkpoint proteins in the superior temporal cortex (STC) of persons with varying severities of AD dementia and compare them to those of cognitively normal controls. To assess whether the CCL changes associated with cognitive impairment in AD are specific to dementia, gene expression of the same proteins was also measured in STC of persons with schizophrenia (SZ), which is also characterized by mitochondrial dysfunction. The expression of CCL-checkpoint and DNA damage response genes: MDM4, ATM and ATR was strongly upregulated and associated with progression of dementia (cognitive dementia rating, CDR), appearing as early as questionable or mild dementia (CDRs 0.5–1). In addition to gene expression changes, the downstream target of ATM-p53 signaling - TIGAR, a p53-inducible protein, the activation of which can regulate energy metabolism and protect against oxidative stress was progressively decreased as severity of dementia evolved, but it was unaffected in subjects with SZ. In contrast to AD, different CCL checkpoint proteins, which include p53, CHEK1 and BRCA1 were significantly downregulated in SZ.

**Conclusions:**

These results support the activation of an ATM signaling and DNA damage response network during the progression of AD dementia, while the progressive decrease in the levels of TIGAR suggests loss of protection initiated by ATM-p53 signaling against intensifying oxidative stress in AD.

## Introduction

Alzheimer’s disease (AD) is the leading cause of dementia in the elderly and is associated with progressive memory loss, cognitive impairment and neurodegeneration. The most prominent characteristics of AD are the presence of amyloid plaques, neurofibrillary tangles and neuronal cell death [1]. The cause(s) of the neurodegeneration in AD is under active debate and study. The most studied molecules implicated in the pathogenesis of AD are derived from its neuropathological hallmarks and include the amyloid β peptide (Aβ - amyloid plaques) and hyperphosphorylated *tau* protein (neurofibrillary tangles) [1]. Increasingly, evidence from different studies suggests an association between neuronal cell cycle events (when proliferating cells undergo stages of mitosis and cell division, which have been summarized as progression from G1 phase through S, G2 and M phase to cell cycle exit at G0) and the process of neurodegeneration in AD [2]–[8]. Although, neurogenesis takes place to a limited degree in some areas of the brain throughout the lifespan, the vast majority of neurons in the adult CNS have been considered to be fully differentiated postmitotic cells and have generally been thought to remain indefinitely in a quiescent non-proliferative state. However, knockout animal models of proteins important for suppressing neuronal proliferation have demonstrated that neurons can abandon their quiescent non-proliferative state at the G_0_ phase and re-enter the cell cycle (CCL) [9]. Consistent with these observations, changes in the expression of markers of CCL (cyclins D [10] and B [3], [10], [11], CDK4 and p16 [2], CDC2 [12], Ki67 [4], p27 [13], BRCA1 [14], Polo-like kinases [15], CIP1-associated regulator of cyclin B [16], p25 - a cleavage product of p35 -the Cdk5 activator [17] and phosphorylated retinoblastoma (p130) protein [18]) have been noted in cortical neurons of postmortem brain specimens from persons with AD that are uncharacteristic of gene and protein expression in postmitotic cells. Additionally, ectopic sub-cellular redistribution of phosphorylated histone H3 [19], CDK11 (G2/M phase regulator) [20], phosphorylated retinoblastoma proteins [21] and CDK5 [22], [23] has been found in AD affected neurons. Activated DNA replication proteins [11], such as the mini-chromosome maintenance (MCM2) proteins, have also been detected in AD brains, indicating that inappropriate S phase entry and DNA replication may occur during disease progression [24]. Observations in AD of phenotypes such as tetraploidy [11], binuclear neuronal cells [25] and premature centromere separation that are rarely present in the brains of unaffected elderly persons, provided additional support for ongoing DNA replication [26], [27] and raise the possibility of chromosomal instability in neural cells of AD patients. Notably, these CCL events appear during early stages of disease [10], [27].

Experimental evidence has also shown an interaction between the accumulation of AD neuropathology hallmarks - Aβ peptide and hyperphosphorylated tau protein - with the activation of CCL and mitosis. Among these are the increased tau phosphorylation and microtubular destabilization that accompanies mitosis [28] and the dose dependent effects of CCL inhibitors on tau phosphorylation [29]. Aβpeptides also influence CCL re-entry, chromosome missegregation and aneuploidy, and induce abnormal cytoplasmic translocation of CDK5 to the nucleus [30], [31]. Interestingly, loss-of-function of presenilin 1, the protein required for γ-secretase cleavage of APP that has been linked to the majority of familial early-onset cases of AD, has also been shown to potently affect neuronal CCL reactivation in an animal model [32]. Together, these findings suggest that neurons affected in AD exhibit elevated expression of various markers of advance phases of the CCL, the progression of which is tightly controlled by a series of checkpoint mechanisms regulating fidelity of cell division. A large body of evidence from postmortem studies, however, advocates against the execution of the full CCL program in AD affected neurons, suggesting the activation of checkpoint mechanism(s) capable of halting the progression of the CCL prior G2/M phase transition, when the cell is prepared for division after completion of DNA replication at the S-phase [33].

Ataxia-telangiectasia mutated (ATM) gene, a serine/threonine kinase is one of the well-characterized cell cycle checkpoint proteins, the substrates of which are involved in double-stranded DNA break responses [34], [35]. Ataxia telangiectasia (A-T) is a human disease caused by ATM deficiency and characterized by the failure of CCL checkpoints, predisposition to cancer, immunodeficiency and by neurologic abnormalities caused by significant loss of neurons [34]. A recent study revealed that ATM can serve as a functional component of, and effector in, cellular redox sensing [36]. ATM and its downstream effector, p53 are not only involved in CCL regulation and tumor suppression, but also in regulating rates of oxidative phosphorylation and glycolysis. The p53-inducible protein, TP53-induced glycolysis and apoptosis regulator (TIGAR), functions to coordinate CCL arrest, apoptosis, glycolysis, and protection against oxidative stress [37], [38].

To study the potential dysregulation of CCL in AD and to map the relationship between CCL checkpoint dysregulation and the progression of AD, we characterize the gene expression of several checkpoint proteins (ATM-ataxia telangiectasia mutated, TP53-tumor protein p53; ATR-ataxia telangiectasia mutated and RAD3 related; ABL1-Abelson murine leukemia viral oncogene homolog 1; CHEK1- checkpoint, S. pombe homolog; MDM4-mouse doubled minute 4 homolog; NBN-nibrin and BRCA1-breast cancer 1 gene) in postmortem brain samples from persons with varying severities of AD-dementia and compare them to cognitively normal controls. We additionally report dementia related changes in the expression of TIGAR and show its neuronal localization. To access disease-specificity of the CCL-associated changes in AD-dementia their expression levels were measured in the postmortem brain of similarly aged persons with schizophrenia, a disease where CCL and oxidative stress related changes have also been reported [39]–[41].

## Results

### Evidence for Cell Cycle Gene Expression Abnormalities during Progression of Dementia from Microarrays

MAS5 normalized samples from 15 cortical regions in the microarray dataset were stratified into the four groups based on CDR score ratings in order to analyze gene expression changes in clinically distinct groups with different severities of dementia and controls. Control subjects were classified as non-demented (CDR = 0). Cases with different severity of dementia were stratified as mild dementia, which includes persons with CDRs of 0.5 or 1, moderate dementia (CDR = 2) and those with CDR scores of ≥3 were categorized as severely demented and combined in a single group. These groups were subjected to contrast analysis [42] in order to rank the strength of gene expression alterations and assess their significance at different stages of dementia relative to the non-demented group (CDR0). This analysis results in sets of differentially expressed genes (with *p≤*0.01; ranging from 3,912–11,322 genes per CDR group) with corresponding individual t-score values, which were uploaded to the MetaCore database and software suite (Thomson Reuter) and subjected to the multiple experiments comparison analysis according to the MetaCore guidelines. Most relevant common and unique gene networks identified from the 3 dementia groups are shown in Table 1. Mitotic cell cycle checkpoints relevant to DNA damage response appeared among the highest scored common gene networks (between all dementia groups, G-score = 37.96; p = 1.67e-5) as well as among unique gene networks for the mild (CDR0.5-1; G-score = 37.53, p = 1.16e-5) and severe dementia (CDR3-5; G-score = 40.68, p = 3.76e-4) groups.

**Table 1:** Most relevant common and unique gene networks in the STG (BA22) derived from MetaCore analysis by comparing groups of cognitively impaired individuals with cognitively intact controls (CDR = 0).

Network processes (Common across all dementia groups)	Size^a^	Pathways^b^	G-scores^c^(*p_s_*<1.0e-4)
**Mitotic cell cycle checkpoint (18.8%),** protein modification by small protein conjugation (27.1%),cellular protein metabolic process (54.2%), **cell cycle checkpoint** **(20.8%)**	50	10	37.96
ATP hydrolysis coupled proton transport (44.9%), energy coupled proton transport, againstelectrochemical gradient (44.9%), ferric iron transport (46.9%)	50	0	21.18
**Unique for mild dementia (CDR = 0.5-1)**			
Canonical Wnt receptor signaling pathway (46.9%), positive regulation of transcription, DNA-dependent(79.6%), positive regulation of RNA metabolic process (79.6%)	50	502	634.83
**Regulation of cell cycle (34%); cell cycle checkpoints** **(22%); regulation of cell cycle arrest (22%)**	50	7	37.53
**Unique for moderate dementia (CDR = 2)**			
Antigen processing and presentation of peptide or polysaccharide antigen via MHC class II (42.9%),interferon-gamma-mediated signaling pathway (44.9%), innate immune response (65.3%)	50	190	245.35
Microtubule-based process (22.9%), intracellular transport of viral proteins in host cell (8.3%), symbiont intracellular protein transport in host (8.3%)	50	0	19.90
**Unique for severe dementia (CDR = 3-5)**			
**Regulation of cell cycle arrest (30.6%), cell cycle checkpoint (28.6%), G2/M transition of** **mitotic cell cycle (24.5%), response to DNA damage stimulus (36.7%), regulation of cell** **cycle process (32.7%)**	50	17	40.68
Enzyme linked receptor protein signaling pathway (68.0%), positive regulation of response to stimulus(76.0%), anatomical structure morphogenesis (86.0%), positive regulation of metabolic process (86.0%)	51	10	21.39

a Size = number of selected nodes (genes);

b Pathways = number of MetaCore pathways recognized within network and.

c G-score = ranks gene networks and based on the enrichment of expressed genes within the network, which is additionally modified with the saturation of the canonical pathways.

Cell cycle related networks highlighted in bold font.

**Figure 1:**
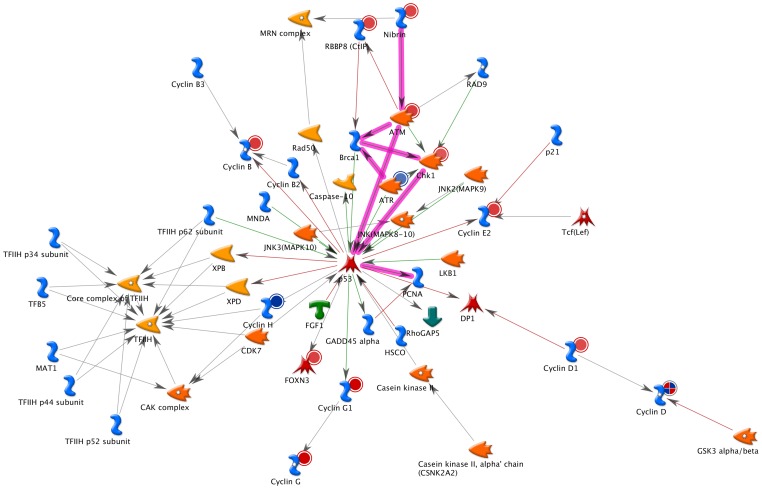
The mitotic cell cycle checkpoint gene network generated by the MetaCore and overlaid with the color coded gene expression changes during dementia. (red – upregulation; blue – downregulation; combination red/blue – differential effect at various stages of dementia). Highlighted is ATM/ATR regulation of G1/S transition pathways. The tumor protein p53 is a hub gene in this network.

Overall, the CCL related gene ontology categories were represented by 832 probe sets targeting 293 genes, which were differentially expressed in groups with varying degrees of dementia severity (p≤0.01, across all CDR groups) compared to controls. In order to understand possible interactions between differentially expressed genes enriched in the CCL related categories, we built potential networks of CCL genes using MetaCore software. As shown in figure 1 the top scoring network (g-score 76.51) consisted of at least 11 signaling pathways implicated in the cell cycle. The main converging and diverging hub gene of this network was tumor protein p53 (TP53). Majority of the pathways within this network were related to DNA damage response and G1/S phase transition (all *p*<1E-14) with the upstream converging nodes including the following genes: ATM, ATR, BRCA1, CHEK1 and NBN, and thus implicating DNA damage ATM/ATR regulation of G1/S transition pathway in the progression of dementia.

### qPCR Analysis of Gene Expression of CCL and DNA Damage Response Genes in the STG during Progression of Dementia

**Figure 2:**
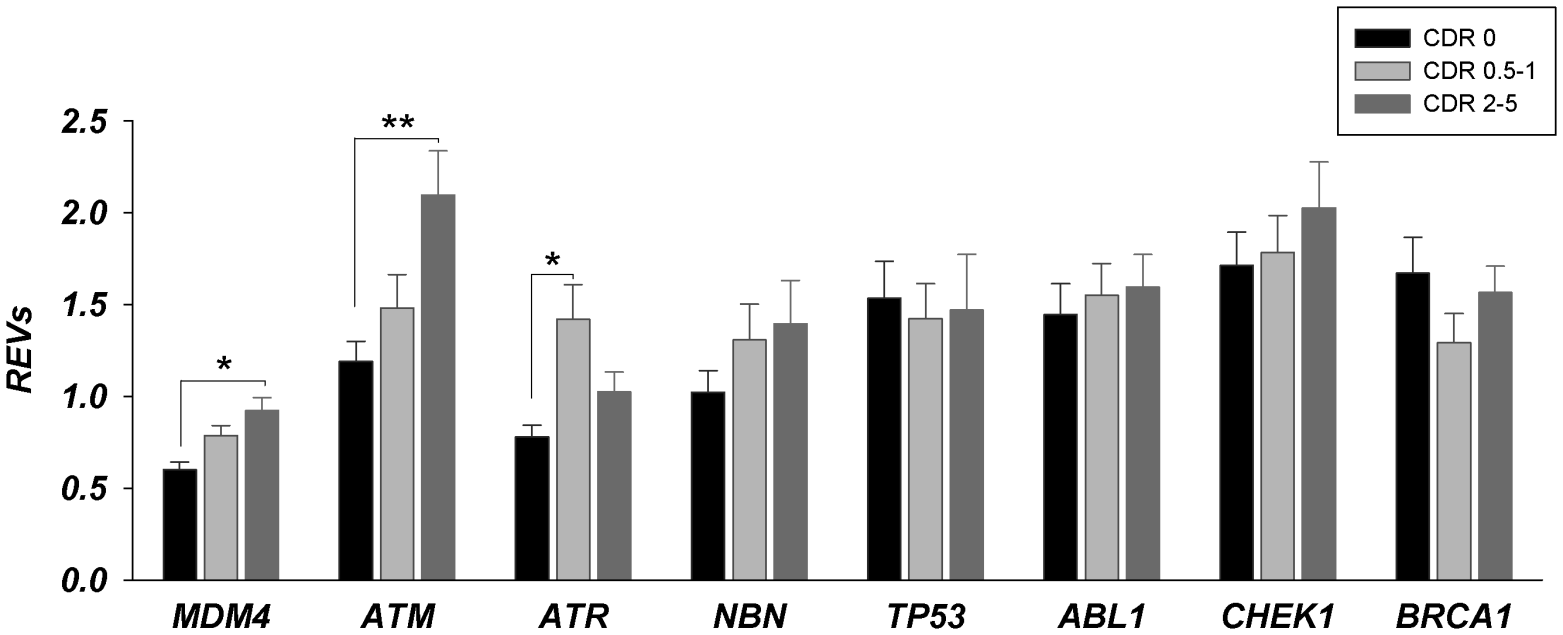
Gene expression changes for ATM/ATR signaling pathway in STG (BA22) during progression of dementia (as a function of CDR). MDM4, ATM and ATR gene expression levels were significantly different in questionable-mild (CDR 0.5-1, light gray bars) and severe (CDR 2-5, dark gray bars) dementia groups relative to control group (black bars). REVs- relative expression values; * -p<0.05; ** -p≤0.01.

Differentially expressed genes in the microarray analysis that encode critical proteins involved in ATM-ATR signaling in response to DNA damage: ataxia telangiectasia mutated (ATM) and RAD3 related (ATR); Abelson murine leukemia viral oncogene homolog 1 (ABL1); checkpoint homolog (CHEK1); mouse doubled minute 4 homolog (MDM4); nibrin (NBN); tumor protein p53 (TP53) and breast cancer 1 gene (BRCA1) were selected for conformational qPCR analysis in the STG of an independent cohort of cases with varying severity of AD dementia, SZ and cognitively normal controls. Comparison of individuals without dementia (NL = CDR 0), with questionable-mild (CDR 0.5-1) and with moderate to severe dementia (CDR 2-5) showed higher levels of MDM4, ATM and ATR gene expression in individuals with dementia (F_2, 112_ = 4.037, p = 0.02 (MDM4); F_2,112_ = 4.357, p = 0.015 (ATM) and F_2,112_ = 3.038, p = 0.052 (ATR); see Figure 2). Comparisons of individuals with and without AD-associated neuropathology also showed high levels of MDM4 and ATM gene expression as a function of increasing neuritic plaque (NP) density (F_3,112_ = 3.601, p = 0.016 and F_3,112_ = 4.802, p = 0.009, respectively) and Braak neuropathological stages (F_4,112_ = 3.042, p = 0.020 and F_4,112_ = 2.816, p = 0.029, respectively). Changes in ATR gene expression as a function of NP density or Braak neuropathological stages were not significant, but showed nominal increases. These results suggest that expression of MDM4, ATM and ATR genes is dysregulated in the earliest recognizable stages of AD-dementia. Levels of MDM4 and ATM were also upregulated early during progression of AD-associated neuropathology and remain elevated throughout the course of AD. Partial correlations of MDM4 and ATM gene expression controlling for Age, pH and PMI demonstrated significant associations with CDR (r = 0.238, df = 107, p = 0.013 for MDM4; r = 0.258, df = 107, p = 0.006 for ATM). In ANCOVAs controlling for age, brain tissue pH, and PMI, only ATM mRNA expression showed significant associations with CDR (F_2,112_ = 3.048, *p = *0.013), NP density (F_3,112_ = 3.008, *p = *0.009) and Braak neuropathological stages (F_4,112_ = 3.059, *p = *0.006). MDM4 and ATR gene expression did not show significant association with NP density and Braak neuropathological stages.

**Figure 3:**
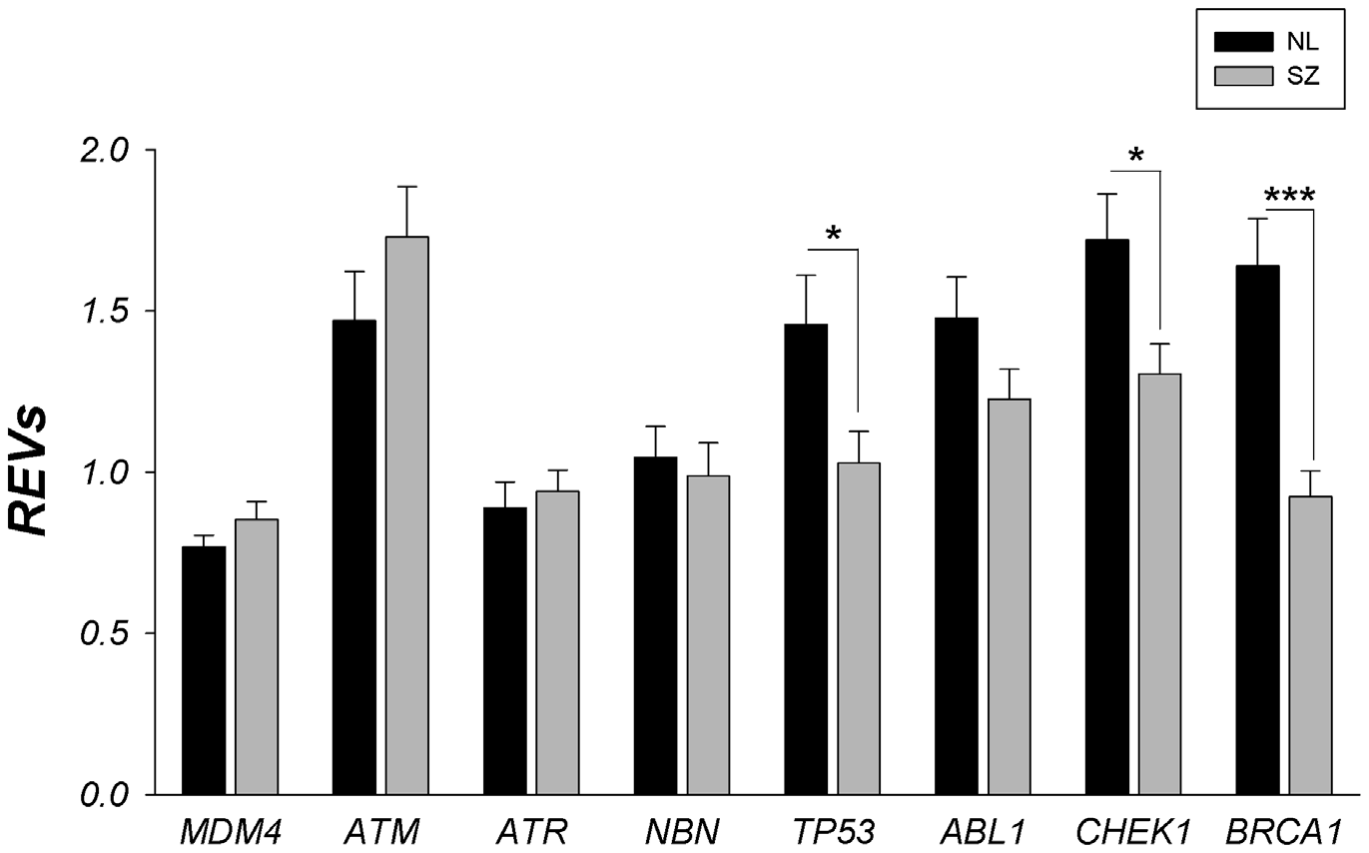
Gene expression changes for ATM/ATR signaling pathway in STG (BA22) in schizophrenia (gray bars) compared to controls (black bars). REVs- relative expression values; NL- controls; SZ- schizophrenia; * - p<0.05; *** - p<0.001.

Comparison of cognitively normal individuals (NL) and individuals with SZ showed lower levels of BRCA1, TP53 and CHEK1 gene expression in individuals with SZ (F_1,120_ = 18.69, p = 2E-4 (BRCA1); F_1,120_ = 5.79, p = 0.018 (TP53) and F_1,120_ = 5.98, p = 0.016 (CHEK1), see Figure 3). Gene expression of MDM4, ATM and ATR were not altered in individuals with SZ compare to controls. Sample pH, PMI, RNA integrity number (RIN) and age of the donors did not correlated significantly with gene expression levels.

### TIGAR Protein Expression during Progression of Dementia

**Figure 4:**
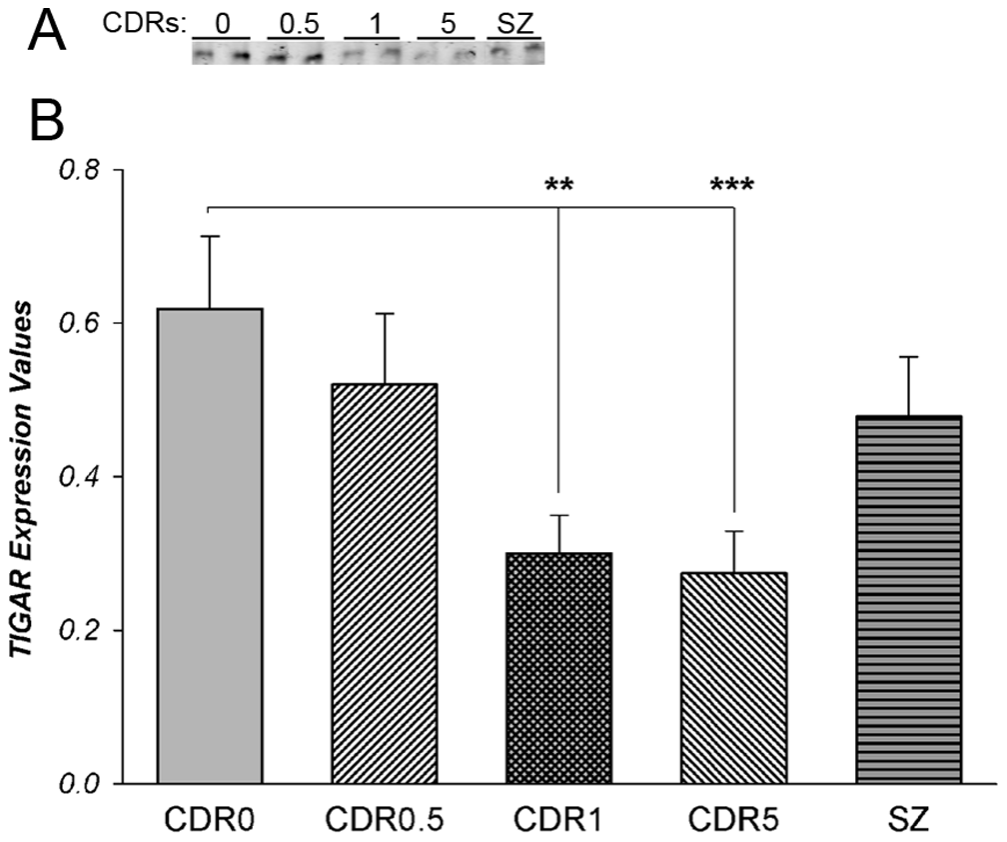
Western blot (A) and protein expression levels of TIGAR (B) in STG (BA22) in dementia (CDRs >0.5) and schizophrenia. TIGAR expression values were normalized to GAPDH. **-p≤0.01; ***-p≤0.001.

To further elucidate responses induced by ATM signaling we evaluated gene expression changes for the broad range of downstream effectors of this pathway in the microarray dataset. Microarray data showed that gene expression of one of these effectors, TIGAR (C12orf5 gene), a p53-inducible gene which regulates the complex balance of ROS-inducing and ROS- decreasing signals propagated by p53 [38] was significantly reduced as dementia severity increased (t-score = –4.9, *p*<0.01). Using Western blotting, we measured protein levels of TIGAR in homogenates from STG of cognitively normal and cognitively impaired persons with varying degrees of severity and from individuals with SZ (Figure 4). Western blot analysis revealed robust TIGAR protein expression in tissue homogenates from STG. Partial correlations of TIGAR protein expression normalized to GAPDH protein levels controlling for age, pH and PMI showed significant negative associations with CDR (r = –0. 420, df = 43, *p = *0.004), but not with Braak scores (r = –0.103, df = 43, *p = *0.497) or NP density (r = –0.230, df = 43, *p = *0.124). Comparison of individuals without dementia (NL = CDR 0), to groups with increased severity of dementia from questionable (CDR 0.5), mild (CDR 1) to severe dementia (CDR 5) showed significant decrease of TIGAR protein expression (Figure 4) in individuals with dementia (F_3,46_ = 3.66, *p = *0.02). ANCOVAs corrected for age, PMI and tissue pH did not affect the significant association with CDR (F_3,46_ = 3.79, *p = *0.006). Significantly less TIGAR protein expression was observed in cases with dementia (CDR ≥1) relative to controls (CDR = 0) (F_1,32_ = 8.51, *p = *0.001). However, comparisons of individuals with and without AD-associated neuropathology did not show significant changes of TIGAR expression either as a function of NP density (F_2,46_ = 2.28, *p = *0.114), or Braak scores (F_4,46_ = 0.66, *p = *0.62). These findings suggest strong downregulation of TIGAR expression associated with measures of dementia severity but not conventional measures of AD neuropathology (NP density and Braak scores). Comparison of cognitively normal individuals (NL) and individuals with SZ showed no difference for TIGAR protein levels in SZ (F_1,34_ = 0.82, p = 0.89).

### TIGAR Immunohistochemistry in Human Temporal Cortex

**Figure 5:**
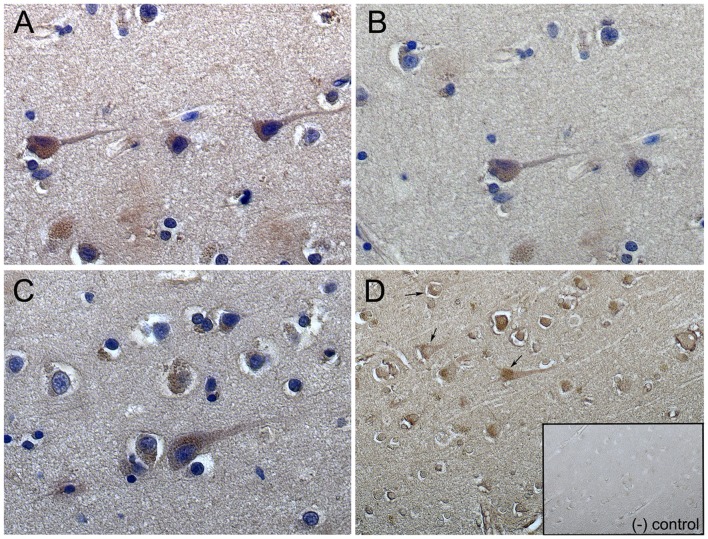
TIGAR is abundant in large pyramidal neurons in deep cortical layers (V–VI) of STG from the human brain. (A–C) Immunostaining for TIGAR visualized by peroxidase substrate DAB (brown staining) and counterstained with hematoxylin to visualize nuclei (blue). (D) Single staining with TIGAR; insert -negative control (secondary antibody staining). Strong staining of TIGAR was prevalent in cytoplasm and sometimes shows nuclear or perinuclear localization in large neurons as indicated by arrows (D).

As TIGAR expression in specialized brain cells has never being examined before, we studied localization of TIGAR protein in human cerebral cortex using immunohistochemistry. Human brain tissue sections from STG containing both grey and the underlining white matter were subjected to immunocytochemistry for TIGAR protein (Figure 5). Large pyramidal neurons in deep cortical layers (V-VI) showed abundant staining for TIGAR, which was prevalent in cytoplasm. Occasionally, TIGAR protein showed nuclear or perinuclear localization in the large neurons as indicated by arrows (Figure 5D). A much weaker TIGAR staining was evident in perinuclear space in oligodendroglia compare to neurons.

## Discussion

This study documents gene and protein expression changes for markers associated with CCL activation indicative of cell cycle re-entry in the superior temporal gyrus during the progression of AD-dementia. Many of these CCL-associated changes occur during the early stages of the development of dementia and typical AD-neuropathology. ATM signaling is essential for the CCL checkpoint mechanism that ensures DNA integrity and repair [35], [43]–[45]. Expression of ATM and some of its downstream effectors was increased during progression of dementia and with increasing severity of AD neuropathology in the grey matter of the STG. It is generally accepted, that accumulation of DNA damage and its impaired repair mechanism is a prominent feature of aging in the CNS [46], and the impairment of this process is believed to be exacerbated in dementia and AD [47]–[49]. There is also growing evidence for the association between DNA damage and increased expression of CCL markers in AD [10], [12]. ATM elicits responses to DNA double-strand breaks and its repair via variety of downstream effectors, including TP53. Activation of ATM signaling begins by the autophosphorylation of ATM dimers, which in turns induces its disassociation into active ATM monomers and promotes DNA damage responses by phosphorylating downstream effectors, including TP53. We measured time-depended stability of the phosphorylation levels of both ATM and TP53 in protein extracts from mouse brain (in the presence of the phosphatase and proteinase inhibitors) and determined that the ATM and TP53 phosphorylation levels decay rapidly during the first 6 hours postmortem (unpublished data), making the determination of their levels unreliable in human postmortem tissue. Nevertheless, consistent with the data presented here, a recent study using immunostaining with a phospho-ATM specific antibody demonstrated that the number of phospho-ATM positive hippocampal neurons (in individuals with mild cognitive impairment), or phospho-ATM positive cerebellar dentate neurons (in definite AD cases - Braak stage V and higher) is increased in cases with dementia compared to controls [50]. These increases paralleled increased phosphorylation of several ATM-specific substrates detected in the same regions from the corresponding cases [50] suggesting ample ATM activation in brain regions vulnerable to neurodegeneration in AD and in mild cognitive impairment.

Although previous analyses of postmortem AD brains have revealed increased p53 expression in overlapping populations of cortical neurons, and cortical and white matter glial cells in regions damaged by neurodegeneration [51]–[53], we found no significant differences in TP53 gene expression in the STG, one of the most vulnerable regions in AD, in individuals stratified by increasing severity of AD dementia or AD neuropathology. On the other hand, the TP53 target gene, TIGAR (p53 induced glycolysis and apoptosis regulator) which encodes protein with structural similarity to the bifunctional enzyme - fructose-2,6-biphosphotase, can hinder progression of glycolysis by conveying carbon metabolism to the pentose phosphate pathway shunt [38]. Therefore, TP53 by activating TIGAR can cause inhibition of glycolysis, and its diversion to the pentose phosphate pathway to maintain sufficient levels of reducing molecules and to protect against DNA-damage induced apoptosis. Our findings indicate that TIGAR protein levels were decreased in various stages of AD dementia severity, suggesting diminishing effect of ATM-p53 signaling in counteracting cell death induced by glycolysis/OXPHOS. The progressive decrease of TIGAR expression reported here is in agreement with the findings of altered post-translational modification of TP53, which result in increased formation of functionally inactive TP53 monomers and dimers, but not functionally active TP53 tetramers in AD brains [54]. Moreover, reported elevated expression of conformationally altered unfolded TP53 in peripheral blood cells from patients with AD [55] raises the question of the impact of protein structural changes on the TP53 activity during progression of dementia. TP53 activates TIGAR under low levels of stress [56]. However, following extended exposure to stress and the induction of the TP53-mediated apoptotic response, TIGAR expression is reduced, suggesting that the induction of the apoptotic response may reflect the loss of protection by the TP53-inducible survival signals [38]. Therefore, TIGAR may play a critical role in the switch of TP53-induced responses to stress and a decrease in its expression may have negative consequences for the survival of cells during progression of dementia. Lack of significant association of TIGAR protein levels with NP density and NFT-related Braak stages also suggests that the regulation of TIGAR expression may be more directly associated with features of AD neurobiology that contribute to cognitive compromise than to neuropathological end-products such as NPs and NFTs.

The changes in the expression of CCL genes observed in AD dementia were specific to AD since they did not generalize to the brains of persons of similar age diagnosed with SZ, the majority of whom were cognitively impaired (average CDR = 2.3). Thus, in contrast to AD dementia, the proteins eliciting DNA damage response signaling were unaffected in the STG of individuals with SZ, whereas some of the downstream key effectors of the ATM signaling cascade associated with the activity of checkpoint proteins were significantly downregulated in SZ. The divergence in the expression of different classes of CCL genes affected in AD and SZ gives credence to the specificity of changes in each disease state and suggests a difference in the etiological substrates of AD and SZ. Given the elevated activity of some CCL indices in the brains of individual with SZ [40], [41], [57], [58], reduced expression of G1/S phase checkpoint proteins (including BRCA1, CHEK1 and TP53) suggests an uninterrupted progression of cell cycle beyond G1-S phases resulting in increased DNA replication, chromosomal instability and increased genetic variability manifested by the presence of somatic polyploid cells with high DNA content. This conclusion is consistent with low-level mosaic aneuploidy in frontal cortex of persons with SZ [59], [60] and elevated DNA synthesis rates in peripheral blood lymphocytes from individuals with SZ compare to normal controls [61]. Given the overall decrease in markers of oxidative metabolism in the frontal cortex of persons with SZ [62], [63] and diminished apoptosis-related DNA fragmentation in elderly individuals with SZ compare to age-matching controls [64], our data may also indicate reduced oxidative damage to DNA as a result of deficiency in energy metabolism..

Taken as whole, the present findings suggest that ATM upregulation and progressive decrease of TIGAR protein levels are distinctive features of dementia progression in AD. Although CCL abnormalities are prevalent in both AD and SZ, these abnormalities are disease specific and distinct from one another. Whether the AD-associated DNA-damage/repair changes reported here are unique to AD or present also in other diseases characterized by eventual neurodegeneration needs to be further elucidated.

In conclusion, ATM may be a crucial link between the damaging effect of ROS and reactivation of mitotic cell cycle in dementia. These data indicate that ATM signaling aimed at reducing the damaging effect of ROS and inducing protective mechanism against DNA damage fails in the cortical grey matter at the earliest stages of AD dementia and may predate the development of the classical neuropathological hallmarks of AD. Futile responses to DNA damage likely further promote progressive deterioration of genome stability and energy metabolism in neural cells.

## Materials and Methods

### Ethics Statement and Brain Specimens

Postmortem brains, donated by the next of kin of deceased subjects participating in studies of aging, early dementia and schizophrenia, were received over a period of 20 years by the Mount Sinai School of Medicine Department of Psychiatry Brain Bank. All assessments were approved by governing institutional review boards including the Mount Sinai School of Medicine and the next of kin of all tissue donors gave formal written consent for research use of the brain tissue. The specimen handling, neuropathology and diagnostic systems used for classifying human brains have been described extensively [65]–[67]. Briefly, brains were removed as soon after death as possible and were divided in half at mid-level, sagittally. The right half was fixed in paraformaldehyde for neuropathological examination, diagnosis and anatomical studies. The left half was sectioned coronally into 0.8 cm blocks, snap-frozen and stored at −80°C for further dissection. For this study, an approximately 1cc block of grey matter from the snap-frozen superior temporal gyrus was dissected at the level of the red nucleus while still frozen and pulverized in a liquid nitrogen cooled mortar and pestle and divided into 50 mg aliquots.

### Demographics and CERAD Classification of the Dementia Cohort

The demographic characteristics of the study cohorts are shown in Tables S1 and S2. For inclusion in the study of dementia, the 198 study brains were selected from over 1,600 potential specimens if they were free of any discernable neuropathology or if they met CERAD neuropathologic criteria for definite, probable, or possible AD with no other significant neuropathologic, neurological or psychiatric comorbidities, including significant cerebrovascular disease, Lewy body disease, Parkinson’s disease or schizophrenia. In each brain, the density of NPs was determined (modified Bielschowsky stain and Aβ immunohistochemistry (clone 6F/3D, Dako Corp., CA)) in CERAD [68] prescribed regions, including the right superior temporal gyrus, from 5 microscopic fields from each of five sections (8 µm thick) and expressed as the number of NPs with amyloid cores per mm^2^. The density of NPs in the superior temporal gyrus (STG, Brodmann area 22) was used in the analyses below. Cognitively intact controls had no known history of any psychiatric or neurologic disorders and no discernable neuropathologic lesions. Table S2 shows the characteristics of the different postmortem cohorts used in the current study. Control subjects had significantly longer postmortem intervals (PMI), nevertheless RNA integrity (RIN ≥7) was good and equal between all comparison groups.

### Demographics of SZ Cohort

The demographic characteristics of the SZ cohort are shown in Table S2. All SZ subjects had been chronically hospitalized at Pilgrim Psychiatric Center (NY) or associated nursing homes for many years. All assessment and postmortem procedures were approved by the Institutional Review Boards of Pilgrim Psychiatric Center, Mount Sinai School of Medicine and the Bronx VA Medical Center. All patients had identical neuropathologic characterization to that described above to rule out discernible neuropathologies such as AD, multi-infarct dementia, etc. [69]. All subjects died of natural causes. Table S2 shows the characteristics of the SZ postmortem cohorts used in the current study. Samples were matched with controls subjects by age and brain pH. Patients with SZ had significantly longer postmortem intervals (PMI), nevertheless RNA integrity (RIN ≥7) was good and equal between all comparison groups.

### Assessment of Dementia and Classification of Subjects into Dementia/AD Neuropathology Severity Groups

The Clinical Dementia Rating (CDR) scale [70]–[72] was used to define the severity or absence of dementia for each case. As previously described [73], a multi-step consensus approach was applied to the postmortem assignment of CDR scores based on cognitive and functional status during the last 6 months of life as described previously [65], [74]. Assignment of CDR included consideration of other measures of cognition, including longitudinally measured MMSE and neuropsychological test performance when available. The CDR assesses cognitive and functional impairments associated with dementia and provides specific severity criteria for classifying subjects as non-demented (CDR = 0) questionably demented (CDR = 0.5), or increasing levels of severity of dementia from CDR = 1 to CDR = 5 (terminal dementia). The qPCR and Western Blotting study cohorts of 173 was stratified into those with and without dementia and those with schizophrenia (Table S1). For pathologic staging of AD neurofibrillary tangle density was assessed using the Consortium to Establish a Registry for Alzheimer's Disease (CERAD) [68], [75] criteria. A modified Bielschowsky method, as described previously [74] was used for neurofibrillary tangle staining and Braak staging. Staging of NFT pathology was based on the criteria by Braak and Braak [76] (Table S1). Braak NFT neuropathology stages were stratified into 5 groups as: 1 = none; 2 = mild transentorhinal (I); 3 = severe transentorhinal (II); 4 = limbic/hippocampal CA1 (III-IV); 5 = isocortical/primary sensory areas (V–VI). Neuritic plaques were counted and specimens were stratified into 4 groups as: 1 = none; 2 = 1–5 per mm2; 3 = 6–10 per mm^2^ and 4 = ≥11 per mm^2^. Neuritic plaque groups reflect a composite score of neuritic plaques counts in 5 cortical regions. Five high power fields (0.5 mm^2^) were examined in each of 5 slides from the cortical region of interest and an average density score was calculated for each region and expressed as mean plaque density per mm^2^. Only neuritic plaques (with and without cores) were included in the NP counts reported here. When plaques were unevenly distributed in each slide, plaques in the region with the highest density were counted.

### RNA Isolation and Microarray Procedure and Data Analysis

The group composition, demographic characteristics and the procedures for RNA isolation and preparation for the microarrays were as described previously [77]. Similarly prepared aliquots from the BA22 (superior temporal gyrus) were used in qPCR [78] and Western blot analyses. Mean RNA integrity numbers for control samples were 7.13±0.90, 7.03±0.89 for dementia groups and 7.02±0.79 for SZ group. Microarray analysis was performed using Affymetrix (Santa Clara, CA) HG-U133AB GeneChip® set as described previously. [79], [80] Statistical comparisons were made using GeneSpring GX12 (Agilent Technologies, Santa Clara, CA). Filtering conditions were a combination of confidence (p≤0.05) and fold change (≥1.4) with Benjamini and Hochberg [81] multiple testing corrections. Microarray dataset used for this study is freely available at https://harouv01.u.hpc.mssm.edu/.

#### RT-qPCR

The mRNA levels of selected cell cycle genes were measured in STG of 173 donors (Tables S1 and S2) by qPCR using TaqMan® probes and primer sets (Table S3) using ABI Prism® 7900HT Sequence Detection System (all from Applied Biosystems). For relative quantification of mRNA expression, relative abundances of the examined genes were calculated using the standard curve method and were further normalized to the geometric means (GMs) of endogenous control-genes as described previously [82]. Three housekeeping genes (RPLOP0, GUSB and PPIA) were used as the endogenous references.

#### Quantitative western blotting

Protein abundance was measured in STG using Western blotting in a subset of randomly selected cases used for qPCR (Tables S1 and S2). Tissue specimens (50mg) were homogenized in Tris/Triton solution: 250 mM sucrose, 50 mM Tris–HCl (pH 7.4), 1 mM EDTA, 2 mM EGTA, 1% Triton X100 containing 1mM PMSF and supplemented with complete cocktails of proteinase/phosphatase inhibitors (Pierce Biotech Inc, Rockford, IL). Total protein concentration in the tissue homogenates was determined with a CBQCA Quantitation Kit (Invitrogen, Carlsbad, CA). Aliquot samples of 15 µg of total protein in duplicate were loaded onto pre-cast 4–20% HEPES-glycine gels from Thermo Scientific Pierce (Rockford, IL, USA) under reduced conditions. A “standard-calibrator” (a mix of small aliquots of tissue from all samples) was used as a calibrator between the gels and run on each gel in duplicate. Blots were incubated with antibodies: rabbit anti- human TIGAR (TP53-induced glycolysis and apoptosis regulator, C12ORF5) was from LifeSpan Biosciences (Seattle, WA); mouse anti-human GAPDH from Meridian Life Science, Inc. (Saco, ME) using SNAP i.d. protein detection system (Millipore Corp., Billerica, MA). Electrophoresis, blotting and infrared (IR) fluorescence detection (IRDye 680 or 800 Goat Anti-appropriate species IgG, Li-Cor Biosciences, Lincoln, NE) were performed under standard conditions. Multiplex western blots were scanned on an Odyssey Infrared Imaging System (Li-Cor Biosciences, Lincoln, NE). The linearity of the dose responses for the antibodies used was established in preliminary experiments. Images were analyzed and quantitated with Odyssey software ver.3 (Li-Cor Biosciences, Lincoln, NE). To account for gel to gel variability, the relative expression values (REVs) of analyzed proteins in each sample was calculated as a ratio between the averaged intensities of the band in the experimental sample and in the “standard-calibrator”. GAPDH was used as loading controls.

### Immunohistochemistry

Human brain tissue was paraffin-embedded and sectioned at 8 µm thickness. All sections were de-paraffinizing with CitriSolv clearing agent (Fisher Scientific, Pittsburgh, PA) for 5 minutes, washed and then soaked in 0.3% hydrogen peroxide to remove endogenous peroxidase activity. Primary antibody - rabbit anti-human TIGAR (LifeSpan Biosciences, Seattle, WA) was diluted in 10% goat serum with 0.5% Tween-20 (1∶50 v/v). Primary antibody staining was detected with biotinylated goat anti-rabbit secondary antibody (1∶400 v/v), avidin-biotin complex horseradish peroxidase (Vector Labs, Burlingame, CA) and peroxidase substrate – DAB/Metal concentrate (Thermo Scientific, Rockford, IL). Some sections were counterstained with Harris modified hematoxylin (Thermo Scientific, Rockford, IL). Stained sections were viewed and photographed at a final magnification x40 using Carl Zeiss Axio Imager Z1 microscope and AxioVision Digital Image Processing System version 4.8.2.

### Statistical Data Analysis

Multiple statistical procedures were employed for different aspects of the study. Max *t*-scores, Pearson correlation coefficients and corresponding p-values (ANOVA) [83], [84] for each individual transcript were calculated by contrast analysis of MAS5.0 normalized microarray data using the GX™ Explorer v.3.0 (Gene Logic Inc., Gaithersburg, MD). t-scores were used as a standardized measure of gene expression change for each individual transcript across all of the analyzed brain regions and described in detail previously [42], [82]. Contrast analysis is an extension of the fold change algorithm which takes into account variability and estimates how well individual gene expression patterns fit a specified model (the contrast pattern vector). The contrast pattern vectors were set up to permit detection of increased expression of genes in the tested sample set if they were present. Accordingly, larger positive scores together with significant (*p≤*0.01) Pearson correlation coefficients meant that the pattern of variation of expression values between sample sets closely follows the pattern represented by the contrast vectors, indicating upregulation of gene expression. Large negative *t*-score values together with significant (*p≤*0.01) negative Pearson correlation coefficients meant that the pattern of variation is the inverse to the pattern represented by the contrast vectors, indicating downregulation of gene expression. Finally, *t-*scores close to zero meant that the gene's expression pattern matches neither the contrast pattern nor its inverse, or that the amount of variation between sample sets was comparable to or smaller than the variation within sample sets. Datasets of differentially expressed genes were subjected to the multiple experiments comparison analysis using MetaCore database and software suite (Thomson Reuter) according to the MetaCore guidelines. Most relevant gene networks with high G-scores were identified in various dementia groups. Highly positive G-scores meant that the networks were saturated with genes from the analyzed gene set and the network contained few to none high degree nodes that were not represented by the analyzed gene set [85]. The *p-*values represent the probability that particular mapping may arise by chance, given the numbers of genes in the set of all genes within the network, genes within a particular network, and genes in the analyzed gene set.

For qPCR experiments, a preliminary analysis assessed linear associations of gene expression with gender, pH and PMI to evaluate their use as covariates. In addition, age, the most significant risk factor for dementia and a critical determinant of the extent of AD associated neuropathology, was used as a covariate in all analyses regardless of its association with the dependent variables. We determined the linear association of gene expression with CDR, Braak stages and NP density by partial correlation analyses, controlling for potential covariates if preliminary analyses showed significant correlation with the gene expression levels under analysis. One way ANOVAs with Tukey’s *post hoc* analysis were used to compare relative mRNA expression of analyzed genes in experimental groups stratified based on their clinical and neuropathology characteristics. ANCOVA was performed for each categorical variable controlling for sex, PMI, RIN and any other potential covariates. Comparison of the demographic variables and relative abundance of TIGAR protein in Western blots was performed using separate one-way ANOVAs. All procedures were performed using SPSS (IBM ver.19).

## Supporting Information

Table S1 **Controls and AD groups’ classifications for gene and protein expression studies.**
(DOC)

Table S2 **Demographic characteristics of study cohorts used for gene and protein expression analyses.**
(DOC)

Table S3 **TaqMan gene expression assays used in the study.**
(DOC)
