# Correction:Cell Cycle Checkpoint Abnormalities during Dementia: A Plausible Association with the Loss of Protection against Oxidative Stress in Alzheimer’s Disease

**DOI:** 10.1371/annotation/59ecb64c-1f53-4d8a-903c-2f835e78bd13

**Published:** 2013-07-17

**Authors:** Pavel Katsel, Weilun Tan, Peter Fam, Dushyant P. Purohit, Vahram Haroutunian

A word was dropped from the title. The full title of this article is:

Cell cycle checkpoint abnormalities during dementia: A plausible association with the loss of protection against oxidative stress in Alzheimer's disease 

