# Supplementary material for: Cycle Checkpoint Abnormalities during Dementia: A Plausible Association with the Loss of Protection against Oxidative Stress in Alzheimer’s Disease
Source: PLoS One. 2013 Jul 5;8(7):e68361. doi: 10.1371/journal.pone.0068361 (PMC3702571; doi:10.1371/journal.pone.0068361)
Supplement: Table S1 — Controls and AD groups’ classifications for gene and protein expression studies. (DOC) [file pone.0068361.s001.doc]

**Table S1.** Controls and AD groups’ classifications for gene and protein expression studies.

| Gene Expression | | | Protein Expression | | |
| --- | --- | --- | --- | --- | --- |
| CDR groups | Dementia severity | Subjects number | CDR groups | Dementia severity | Subjects number |
| 0 | No dementia | 39 | 0 | No dementia | 14 |
| 0.5-1 | Mild dementia | 32 | 0.5 | Questionable dementia | 14 |
| 2-5 | Moderate/severe dementia | 41 | 1 | Mild dementia | 8 |
|  |  |  | 3-5 | Severe dementia | 10 |

| Braak groups | Braak Stages | Subjects number | Braak groups | Braak Stages | Subjects number |
| --- | --- | --- | --- | --- | --- |
| 0 | None | 30 | 0 | None | 14 |
| I | Mild transentorhinal | 15 | I | Mild transentorhinal | 8 |
| II | Severe transentorhinal | 22 | II | Severe transentorhinal | 10 |
| III-IV | Limbic/Hippocampal CA1 | 17 | III-IV | Limbic/Hippocampal CA1 | 7 |
| V-VI | Isocortical/Primary sensory | 28 | V-VI | Isocortical/Primary sensory | 7 |

| NP Density groups | NP Plaques ( per mm^2^) | Subjects number | NP Density groups | NP Plaques ( per mm^2^) | Subjects number |
| --- | --- | --- | --- | --- | --- |
| 1 | 0 | 67 | 1 | 0 | 19 |
| 2 | 1-5 | 22 | 2 | 1-5 | 13 |
| 3 | 6-10 | 10 | 3 | 6-10 | 4 |
| 4 | > 11 | 13 | 4 | > 11 | 10 |
