# Supplementary material for: Cycle Checkpoint Abnormalities during Dementia: A Plausible Association with the Loss of Protection against Oxidative Stress in Alzheimer’s Disease
Source: PLoS One. 2013 Jul 5;8(7):e68361. doi: 10.1371/journal.pone.0068361 (PMC3702571; doi:10.1371/journal.pone.0068361)
Supplement: Table S2 — Demographic characteristics of study cohorts used for gene and protein expression analyses. (DOC) [file pone.0068361.s002.doc]

**Table S2.** Demographic characteristics of study cohorts used for gene and protein expression analyses.

| Characteristics | **NL (CDR0) qPCR** | **MD (CDR0.5-1) qPCR** | **SD (CDR>2)**  **qPCR** | **SZ**  **qPCR** | **NL (CDR0)**  **Western** | **MD (CDR0.5-1) Western** | **SD (CDR>2)**  **Western** | **SZ Western** |
| --- | --- | --- | --- | --- | --- | --- | --- | --- |
| # subjects | 39 | 32 | 41 | 61 | 14 | 22 | 10 | 11 |
| Sex (M/F) | 24/15 | 15/17 | 7/34 | 21/40 | 6/8 | 15/7 | 2/8 | 8/3 |
| Age (years) | 69.8 (2.0)† | 86.9 (2.8) | 87.9 (1.2) | 72.9 (1.5) | 74.3 (2.5) | 80.4 (2.1) | 84.4 (1.8) | 75.6 (3.3) |
| Brain pH | 6.5 (0.05) | 6.5 (0.05) | 6.4 (0.08) | 6.5 (0.03) | 6.5 (0.04) | 6.5 (0.05) | 6.5 (0.05) | 6.5 (0.04) |
| PMI (min) | 1119 (110) | 668 (101) | 370 (43) | 1413 (121) | 649 (120) | 773 (187) | 374 (77) | 1897 (287) |

Data are expressed as mean (SEM). NL- controls (CDR0); MD- mild dementia (CDRs 0.5-1); SD- severe dementia (CDRs >2); SZ- schizophrenia.
