# Supplementary material for: Cycle Checkpoint Abnormalities during Dementia: A Plausible Association with the Loss of Protection against Oxidative Stress in Alzheimer’s Disease
Source: PLoS One. 2013 Jul 5;8(7):e68361. doi: 10.1371/journal.pone.0068361 (PMC3702571; doi:10.1371/journal.pone.0068361)
Supplement: Table S3 — TaqMan gene expression assays used in the study. (DOC) [file pone.0068361.s003.doc]

**Table S3.** TaqMan gene expression assays used in the study.

| Gene symbol | Gene Name (human) | Assay ID (Hs) | NCBI (NM_) | Target Exon |
| --- | --- | --- | --- | --- |
| ATM | ataxia telangiectasia mutated | 01112307_m1 | 000051.3 | 16 |
| MDM4 | Mdm4 p53 binding protein homolog (mouse) | 00159092_m1 | 002393.3 | 3 |
| ATR | ataxia telangiectasia and Rad3 related | 00354807_m1 | 001184.3 | 24 |
| NBN | nibrin | 00159537_m1 | 002485.4 | 1 |
| TP53 | tumor protein p53 | 01034249_m1 | 001126112.1 | 6 |
| ABL1 | c-abl oncogene 1, non-receptor tyrosine kinase | 01104728_m1 | 005157.4 | 8 |
| CHEK1 | checkpoint homolog (S. pombe) | 00967506_m1 | 001114121.1 | 6 |
| BRCA1 | breast cancer 1, early onset | 01556193_m1 | 027676.1 | 22 |
| GUSB | glucuronidase, beta | 99999908_m1 | 000181.1 | 12 |
| PPIA | peptidylprolyl isomerase A (cyclophilin A) | 99999904_m1 | 000291.2 | 4 |
| RPLPO | RPLPO (large ribosomal protein) | 99999902_m1 | 053275.3 | 3 |
